# Elucidation of Molecular Mechanisms of Lipid-Altered Cytotoxicity of TDP-43 Fibrils

**DOI:** 10.1021/acschemneuro.5c00934

**Published:** 2026-01-29

**Authors:** Yana Purvinsh, Mikhail Matveyenka, Dmitry Kurouski

**Affiliations:** Department of Biochemistry and Biophysics, 14736 Texas A&M University, College Station, Texas 77843, United States

**Keywords:** CTD TDP-43, phosphatidylserine, cardiolipin, phosphatidylcholine, qPCR, neurons

## Abstract

Progressive aggregation of TAR DNA-binding protein 43 (TDP-43) is a hallmark of numerous neurodegenerative diseases, including amyotrophic lateral sclerosis, frontotemporal dementia, Alzheimer’s disease, and limbic predominant age-related TDP-43 encephalopathy (LATE). This highly conserved nuclear RNA/DNA-binding protein is involved in the regulation of RNA processing. The C-terminal domain (CTD) of TDP-43 plays a key role in protein solubility, cellular localization, and protein–protein interactions. CTD is rich in glycine, glutamine, and asparagine, which facilitate TDP-43 aggregation into amyloid oligomers and fibrils observed in the brain. In this study, we examine the role of lipid bilayers in the aggregation properties of the CTD of TDP-43. We found that lipid bilayers composed of anionic phosphatidylserine and cardiolipin accelerated TDP-43 aggregation. Although lipids did not alter the secondary structure, they altered the cytotoxicity that TDP-43 fibrils exerted to rat dopaminergic cells. Using molecular methods, we showed that TDP-43 fibrils damage cell endosomes. This causes aggregate leakage into the cytosol, where TDP-43 fibrils impair cell autophagy, simultaneously triggering a severe unfolded protein response in the endoplasmic reticulum. Our results indicate that TDP-43 aggregation may be linked to pathological changes in the lipid profiles of neurons.

## Graphical abstract

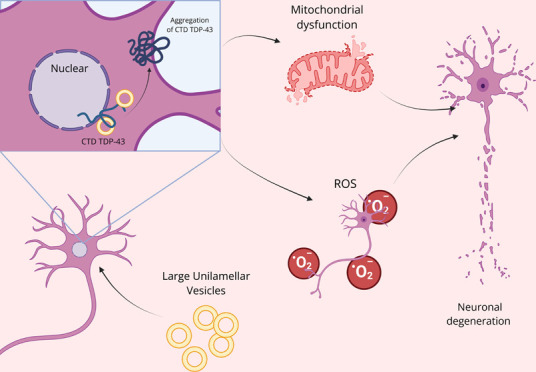

## Introduction

A large number of neurodegenerative diseases, including amyotrophic lateral sclerosis (ALS), frontotemporal dementia (FTD), Alzheimer’s disease (AD), limbic predominant age-related aggregation of TAR DNA-binding protein 43 (TDP-43) encephalopathy (LATE), and Parkinson’s disease, are linked to the TDP-43.^1–6^ In normal cells, TDP-43 is primarily localized in the nucleus, where it is involved in transcriptional regulation and alternative splicing of RNA.^7–9^ Under pathological conditions, TDP-43 accumulates in the cytosol, where it forms stress granules with several other proteins and RNAs.^10–14^ Although molecular mechanisms of TDP-43 aggregation remain unclear, a growing body of evidence suggests protein aggregation is determined by its C-terminus domain (C-terminal domain (CTD)), Figure 1.^15,16^ CTD is essential for protein solubility, cellular localization, protein-RNA, and protein–protein interactions.^17,18^ However, high quantities of glycine, glutamine, and asparagine in CTD make TDP-43 intrinsically unstable.^19,20^ Similar protein-RNA recognition and glycine-rich domains were also observed by Gitler and Shorter in the FUS protein, suggesting that the presence of such domains in proteins determines their aggregation properties.^20^ Furthermore, most mutations associated with ALS and FTD are located in the CTD, whereas the expression of mutant TDP-43 A315T in a *Drosophila* model resulted in an increase in protein aggregation and neurotoxicity.^21–24^

**Figure 1:**
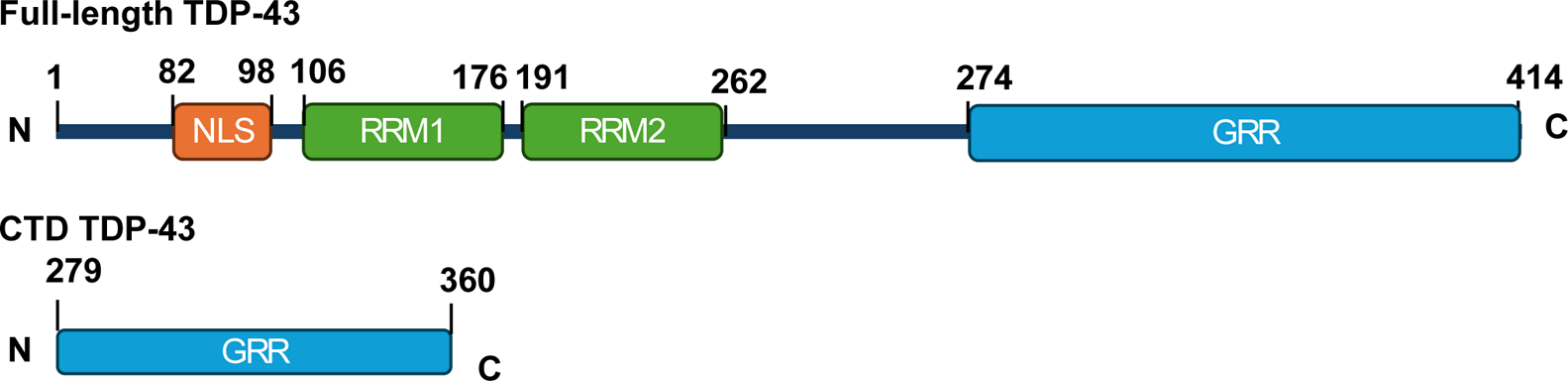
Schematic representation of the structure of full-length and CTD of TDP-43 with nuclear localization signal (NLS) motif, 2 RNA-recognition motifs (RRM1 and RRM2), and a glycine-rich region (GRR).

The mechanism of how TDP-43 spreads across the brain remains unclear.^25,26^ Several research groups demonstrated that TDP-43 aggregates propagate from cell to cell via autophagy.^27,28^ Specifically, Feiler et al. showed the presence of TDP-43 in the microvesicles and exosomes of HEK293 cells transfected with TDP-43. At the same time, Smethurst et al. did not observe TDP-43 aggregates in cells incubated with conditioned medium for 3 days. Once present in the cytosol, TDP-43 aggregates can disrupt nucleocytoplasmic transport and damage nuclear pore complexes.^29,30^ Similar cytotoxic effects are also exerted by amyloid-β­(Aβ), α-synuclein (α-syn), and Tau oligomers and fibrils.^31–34^ Our group demonstrated that the aggregation properties of Aβ, α-syn, and Tau could be altered by lipids that constitute plasma and organelle membranes.^35–38^ For instance, phosphatidylcholine (PC), cardiolipin (CL), and cholesterol accelerated Aβ aggregation and drastically increased cytotoxicity of Aβ oligomers and fibrils.^36^ Dou et al. demonstrated that the presence of lipid vesicles composed of PC and phosphatidylserine (PS) present during protein aggregation drastically enhanced cytotoxicity of α-syn fibrils.^37^ Ali et al. found that cytotoxicity of Tau aggregates, and the mechanisms by which Tau fibrils exert cytotoxic effects, could be altered by PC, PS, and cholesterol.^38^ Corucci and co-workers investigated the interactions between synthetic model phospholipid membranes and a TDP-43 fragment lacking the first 84 N-terminal residues, called M85.^39^ It was shown that the charge of lipids affects the interactions between M85 and membranes. Specifically, negatively charged lipids facilitate M85-membrane interactions and promote protein aggregation. However, the effect of lipids on the CTD of TDP-43 remains unclear. To this end, we investigated the effect of large unilamellar vesicles (LUVs) composed of PC, CL, and PS on the rate of TDP-43 CTD aggregation. We also used atomic force microscopy (AFM) and nano-infrared spectroscopy (atomic force microscopy-infrared (AFM-IR)) to examine the morphology and secondary structure of TDP-43 CTD fibrils. Finally, rat dopaminergic neurons were used to investigate the extent to which lipids alter the cytotoxicity of the TDP-43 CTD fibrils. This multiple approach technique aims to elucidate the effects that lipids play in the rate of TDP-43 aggregation and its subsequent toxicity.

## Methods

### Protein Expression and Purification

*Escherichia coli* BL21 (DE3) cells (New England BioLabs, cat. no. C2527H) were transformed with a plasmid encoding the 6xHis-TDP-43_279–360_ CTD. A single colony from the LB-agar plate was inoculated to 50 mL LB medium containing kanamycin (25 mg mL^–1^) and incubated at 37 °C for 16 h with shaking at 200 rpm. The overexpressed culture was diluted with preculture LB medium into 2 L, protein expression was induced at an OD600 of 0.6 by the addition of 1 mM IPTG (isopropyl β-d-thiogalactopyranoside, Fisher Scientific, cat. no. BP1755-100) and further incubation for 14 h at 16 °C.

Cells from the 2 L culture were harvested by centrifugation at 3000*g* for 15 min at 4 °C. The pellets were then resuspended in 80 mL of lysis buffer on ice (6 M urea (Sigma-Aldrich, cat. no. U5378), 50 mM sodium phosphate buffer pH 8.0, 500 mM NaCl, and 1 mM DTT (Sigma-Aldrich, cat. no. 3483-12-3)) and lysed with the Microfluidizer LM10, followed by another round of centrifugation at 15,000*g* for 45 min at 4 °C. The supernatant was collected, filtered (0.45 μm), and loaded onto Ni-NTA agarose (Invitrogen, cat. no. R901-15) in a gravity flow column, pre-equilibrated with 6 M urea; 50 mM sodium phosphate (pH 8.0), 500 mM NaCl, 10 mM imidazole, and 1 mM DTT. After washing with buffer (6 M urea, 50 mM sodium phosphate buffer pH 8.0, 500 mM NaCl, 50 mM imidazole, and 1 mM DTT), the 6xHis-TDP-43_279–360_ CTD protein was eluted in the same buffer with 300 mM imidazole, with a total elution volume of 20 mL. The eluted fractions containing 6xHis-TDP-43_279–360_ CTD were pooled, analyzed by sodium dodecyl sulfate-polyacrylamide gel electrophoresis, and concentrated using a 3 kDa MWCO centrifugal filter unit (Sigma-Aldrich, cat. no. UFC9003). After concentration, the protein was dialyzed against 2 × 1 L 30 mM sodium phosphate buffer, pH 8.0, 300 mM NaCl, and 2 g/L histidine (Thermo Fisher Scientific, cat. no. A10413.22). Protein concentration was determined by optical absorption spectroscopy using a molar absorption coefficient at 280 nm (ε280) of 5500 M^‑1^ cm^–1^ (molecular weight ∼ 10.4 kDa). Experimental workflows of the following experiments are shown in Figure S1.

### Liposome Preparation

To prepare LUVs, lipids (PC, Avanti, cat. no. 850355; PS, Avanti, cat. no. 840037; CL, Avanti, cat. no. 710333) were initially dissolved in chloroform to ensure complete dissolution. The solvent was then evaporated using dry nitrogen gas, forming a thin lipid film. This film was rehydrated in phosphate-buffered saline (PBS, pH 7.4; Gibco, cat. no. 10010023). After ensuring complete rehydration, the lipid solutions were incubated at approximately 50 °C in a water bath for 30 min, followed by rapid cooling in liquid nitrogen for 3–5 min. This freeze–thaw cycle was repeated ten times to achieve uniform lipid vesicles. To standardize the vesicle size, the solutions were processed using an extruder fitted with a 100 nm pore-size membrane (Avanti, cat. no. 610005). The size distribution of the LUVs was confirmed by using dynamic light scattering, which demonstrated a consistent diameter of 100 ± 10 nm.

### Protein Aggregation

For aggregation experiments, 10 μM CTD TDP-43 protein was in a dialysis buffer consisting of 30 mM sodium phosphate buffer (pH 8.0), 300 mM NaCl, and 2 g/L histidine. In the presence of lipids, the protein was mixed with lipid vesicles in an equimolar ratio. The samples were then transferred to a 96-well plate and incubated at 37 °C for 48 h with continuous agitation at 510 rpm in a plate reader (Tecan Spark, Männedorf, Switzerland). In parallel, control experiments without lipids were conducted under identical conditions to evaluate the effect of lipid interactions on protein aggregation.

### Kinetic Measurements

Protein aggregation kinetics were monitored by using a thioflavin T (ThT) fluorescence assay. For this, samples were prepared by mixing with a 25 μM ThT solution and transferring them into a 96-well plate. The plate was incubated in a plate reader at 37 °C for 48 h with continuous agitation at 510 rpm. Fluorescence readings were collected every minute with an excitation wavelength of 450 nm and an emission wavelength of 488 nm. Each curve represents the average of four independent measurements. Statistical significance was evaluated using one-way ANOVA followed by Bonferroni’s test, performed in GraphPad PRISM (v. 10.2.3, Dotmatics, USA). See Table 1.

**Table 1:** Statistical Analysis Results of Experimental Data

	F(DFn, DFd)	P value	corresponding figure
t_lag_	F(3, 8) = 137.1	*P* < 0.0001	2
*t*_1/2_	F(3, 8) = 28.08	*P* = 0.0001	2
height	F(3, 116) = 6.304	*P* = 0.0005	3
parallel β-sheet	F(3, 8) = 0.8031	*P* = 0.5264	4
random coiling, α-helix, β-turn	F(3, 8) = 1.873	*P* = 0.2124	4
antiparallel β-sheet	F(3, 8) = 2.759	*P* = 0.1117	4
JC-1	F(8, 21) = 88.87	*P* < 0.0001	5
ROS	F(8, 18) = 53.91	*P* < 0.0001	5
Cmpb1	F(8, 21) = 88.87	*P* < 0.0001	6
Gal3	F(8, 21) = 72.86	*P* < 0.0001	6
ATF6	F(6, 14) = 58.87	*P* < 0.0001	7
PERK	F(6, 14) = 187.6	*P* < 0.0001	7
XBP1	F(6, 14) = 17.39	*P* < 0.0001	7
LC3b	F(6, 14) = 29.15	*P* < 0.0001	7
P62	F(6, 14) = 58.22	*P* < 0.0001	7

### Atomic Force Microscopy

The AIST-NT-HORIBA system (Horiba, USA) was employed to examine the topological features of the aggregates. Silicon AFM probes (Appnano, USA) with a force constant of 2.7 N/m and a resonance frequency of 50–80 kHz were used in tapping mode. Prior to imaging, samples were diluted with deionized water and deposited onto precleaned glass coverslips. Image preprocessing and analysis were conducted using AIST-NT software (v3.4.2, Horiba, USA).

### Atomic Force Microscopy-Infrared Spectroscopy

Protein samples were deposited onto a 70 nm gold-coated silicon wafer in volumes of 3–6 μL. Samples were allowed to air-dry for 15–20 min, followed by rinsing with deionized water and drying under a nitrogen stream. AFM-IR imaging and spectra were acquired by using a NanoIR3 system (Bruker, USA) equipped with a QCL laser. Contact-mode AFM tips (ContGB-G probes, NanoAndMore) were utilized, with optimization performed using a poly­(methyl methacrylate) standard for wavenumbers ranging from 1400 to 1800 cm^–1^. Imaging parameters included scan rates of 0.3–0.8 Hz, dimensions of 1–10 μm, and resolutions of 256 points in *X* and *Y* axes. Spectral acquisition involved collecting an average of 30 spectra per sample with coaveraging of three per spectrum.

### JC-1 Assay

Mitochondrial membrane potential was assessed by using the JC-1 assay. N27 cells (Millipore, cat. no. SCC048, RRID:CVCL_D584) were cultured in RPMI-1640 medium (Thermo Fisher Scientific, cat. no. 11875085) supplemented with 10% fetal bovine serum (FBS; Thermo Fisher Scientific, cat. no. A5209501) and Normocin antibiotic (InvivoGen, cat. no. ant-nr-1) at 37 °C in 5% CO_2_. The N27 cell line is not listed as commonly misidentified by ICLAC. The cells were not authenticated post-purchase and were used for a maximum of 10 passages. Cells were seeded at a density of 20,000 cells per well in flat-bottom 96-well plates (83.3924.300, Sarstedt, Germany) and incubated for 24 h. Following a 24 h treatment with protein samples, cells were collected, centrifuged at 400*g* for 5 min, and resuspended in PBS. JC-1 dye from the MitoProbe JC-1 Assay Kit (Thermo Fisher Scientific, cat. no. M34152A) was applied at 2.0 μg/mL, and cells were incubated for 30 min at 37 °C. Mitochondrial depolarization was determined relative to a positive control treated with carbonyl cyanide m-chlorophenyl hydrazone at a final concentration of 50 μM. After centrifugation at 400*g* for 5 min, cells were resuspended in PBS and analyzed using a LSR II flow cytometer (BD, USA), using red and green fluorescence channels to detect ΔΨm changes. Measurements were conducted in triplicate.

### ROS Assay

The reactive oxygen species (ROS) assay was employed to measure the intracellular levels of ROS. N27 cells were cultured as described above until 70–80% confluence. After 24 h treatment with protein samples, ROS detection reagent (Thermo Fisher Scientific, cat. no. C10422) was added to a final concentration of 5 μM. Cells were incubated with the ROS reagent for 30 min at 37 °C in 5% CO_2_, washed with PBS and resuspended in 200 μL of PBS. For ROS, positive control cells were treated with menadione at a final concentration of 200 μM for 30 min. Fluorescence was measured on a flow cytometer using the red channel (λ = 633 nm). All measurements were carried out in triplicate.

### Quantitative Polymerase Chain Reaction

Total RNA was isolated using the GeneJET RNA Purification Kit (Thermo Scientific, cat. no. K0732), and RNA concentration was measured using a NanoDrop One spectrophotometer (Thermo Fisher Scientific, USA). Next, synthesis of cDNA was performed with the High-Capacity cDNA Reverse Transcription Kit (Thermo Fisher Scientific, cat. no. 4368814). Specific primers were designed for the following genes: **p62**, **LC3**, **PERK**, **ATF6**, and **XBP1** (primer sequences listed in Table S1, Zhaliazka et al., 2024). Quantitative polymerase chain reactions (qPCRs) were carried out on a QuantStudio 7 Flex Real-Time PCR System (Thermo Fisher Scientific, USA), using Luna Universal qPCR Master Mix (New England Biolabs, cat. no. M3003) in 96-well optical plates (Thermo Fisher Scientific, cat. no. A36930). Each reaction included gene-specific primers, cDNA, and master mix. GAPDH was used as the housekeeping gene, and 40 amplification cycles were performed.

### Fluorescence Microscopy

N27 cells were seeded (10,000 cells/well) in 96-well glass-bottom plates (Cellvis, cat. no. P961.5HN) in RPMI-1640 medium with 10% FBS and incubated overnight at 37 °C and 5% CO_2_. Next, the cell culture medium was replaced with fresh RPMI-1640 Medium containing 5% FBS and including the protein samples. Plates were incubated at 37 °C for 24 h. Next, ROS and JC-1 reagents were added to achieve final concentrations of 5 μM. Cells were incubated for 20 min at 37 °C in a 5% CO_2_ and stained with NucBlue Live Cell Ready Probes (Thermo Fisher Scientific, cat. no. R37605). Imaging was performed using the EVOS M5000 Imaging System (Thermo Fisher Scientific, USA) equipped with an Olympus UPlanApo 100*x*/1.35 oil iris ∞/0.17 objective and blue, green, and deep red filters. Experiments were conducted in triplicate.

### Membrane Leakage Assay

To assess membrane integrity and autophagic responses, N27 cells were transfected with plasmids coding for Chmp1b (membrane repair), Gal3 (autophagy marker), and TFEB (lysosomal biogenesis regulator) using GeneX Plus transfection reagent (ATCC, cat. no. ACS-4004). After cell reached ∼ 70% confluency, transfection was performed in serum-free DMEM (Corning, cat. no. 10-013) for 8 h. Next, TDP-43 aggregates were added to the cells and incubated for 24 h at 37 °C in a 5% CO_2_. Fluorescent imaging was carried out using an EVOS M5000 Imaging System. All experiments were performed in triplicate.

### Data Analysis

The quantification of the relative expression of genes was determined using the comparative Ct method (2^–ΔΔCt^). In this approach, ΔCt represents the difference in threshold cycles between the target gene and the housekeeping gene, while ΔΔCt represents the difference in ΔCt values between the cells exposed to amyloid samples and the control (buffer). Real-time PCR data (ΔΔCt method) were processed in Microsoft Excel for Microsoft 365 (Microsoft Corporation, USA). Statistical significance of reported results was analyzed using one-way ANOVA followed by Bonferroni’s test, performed in GraphPad PRISM (v. 10.2.3, Dotmatics, USA) (Table 1).

For JC-1 and ROS assays, data were calculated relative to the positive control and were processed with BD FACSDiva software (version 8.0.1, BD, USA).

Prior to AFM-IR data analysis, spectral artifacts in the 1646–1652 cm^–1^ range, arising from chip transitions, were removed by “zapping”. The spectral resolution was 2 cm^–1^ per point, and data were processed using MATLAB (v. R2022a, MathWorks, USA), employing Savitzky–Golay smoothing (0 polynomial order) and area normalization. Baseline corrections (level + zero) and peak fitting were applied using GRAMS/AI software (v. 9.3, Thermo Fisher Scientific, USA).

For the statistical analyses of all the data discussed above, one-way ANOVA followed by Bonferroni’s test was used (Table 1). Prior to this, assessment of data normality was performed using the Shapiro–Wilk Test. Not a single data point was excluded from the manuscript; no tests for outliers were conducted. Statistical analyses were performed in GraphPad PRISM software.

## Results and Discussion

Our results show that in the lipid-free environment, CTD TDP-43 aggregated, exhibiting a short lag-phase (60 ± 0.2 min) that was followed by a rapid increase in ThT fluorescence, Figure 2 and Table 1. Zwitterionic PC LUVs slightly elongated the lag-phase (61 min ± 0.1 min), while anionic PS (32 min ± 0.3 min) and CL (39 min ± 0.5 min) caused a decrease in the length of the CTD TDP-43 lag-phase. These results indicated that lipids accelerated CTD TDP-43 aggregation. Our results also indicated that the lipid net charge strongly influenced CTD TDP-43 aggregation. Specifically, anionic lipids had a much stronger acceleration effect compared to zwitterionic lipids. These results indicate that anionic lipids interact with positively charged amino acids located in the CTD of TDP-43. Similar conclusions could be made about the rate of fibrillar growth. We found that both CL and PS enhanced the rate of fibril formation compared to the lipid-free condition, while the effect of PC LUVs was statistically insignificant. Based on these findings, we could conclude that LUVs altered both CTD TDP-43 nucleation and fibril formation.

**Figure 2:**
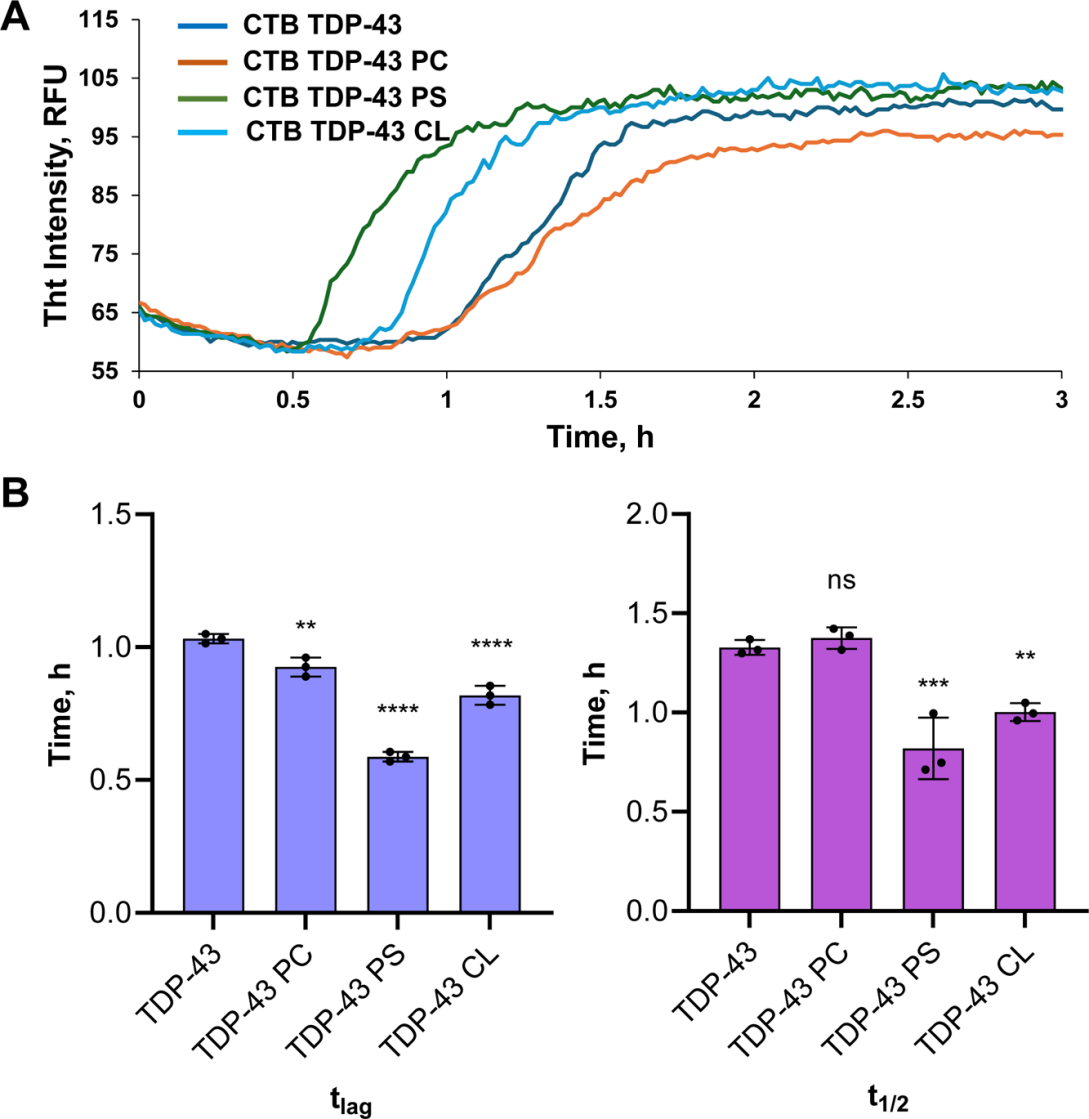
Lipids facilitate CTD TDP-43 aggregation. ThT kinetics (A) and a bar graph (B) of CTD TDP-43 aggregation in the absence of lipids (CTD) and in the presence of equimolar concentrations of LUVs composed of PC, PS, and CL. The lag-phase (*t*_lag_) corresponds to 10% and half-time (*t*_half_) to 50% of the maximal ThT intensity. Each curve shown in panels is the average of three replicates (*n* = 3). The graphical data are presented as the mean ± SEM. According to one-way ANOVA, **P < 0.01; ***P < 0.001; ****P < 0.0001. NS is a nonsignificant difference.

Similar observations were reported for other amyloidogenic proteins and peptides. For instance, Matveyenka and co-workers showed that anionic lipids caused much greater acceleration of insulin aggregation compared to zwitterionic lipids.^40^ Zhang and co-workers made the same observations for amylin that were confirmed by research results reported by our group.^41,42^ Zhaliazka and co-workers demonstrated that zwitterionic PC and anionic CL accelerated aggregation of amyloid β_1–42_.^36^ However, the acceleration effect was stronger for CL than PC. The same conclusions were made by Lee and Vendruscolo groups for α-syn.^43–47^

Morphological analysis of CTD TDP-43 fibrils formed in the absence of lipids revealed the presence of long, twisted fibrillar species with heights ranging from 5 to 12 nm, Figure 3. Morphologically similar aggregates were observed in other samples. However, none of those fibrils had a clear visible twist, while their height was slightly smaller (4–9 nm). Thus, we could conclude that lipids altered the height of CTD TDP-43 fibrils. It should be noted that beyond height differences and the presence or absence of the twists on the fibrillar surface, we did not observe differences in fibril length distribution, branching, bundling, or presence of oligomeric species between all four samples.

**Figure 3:**
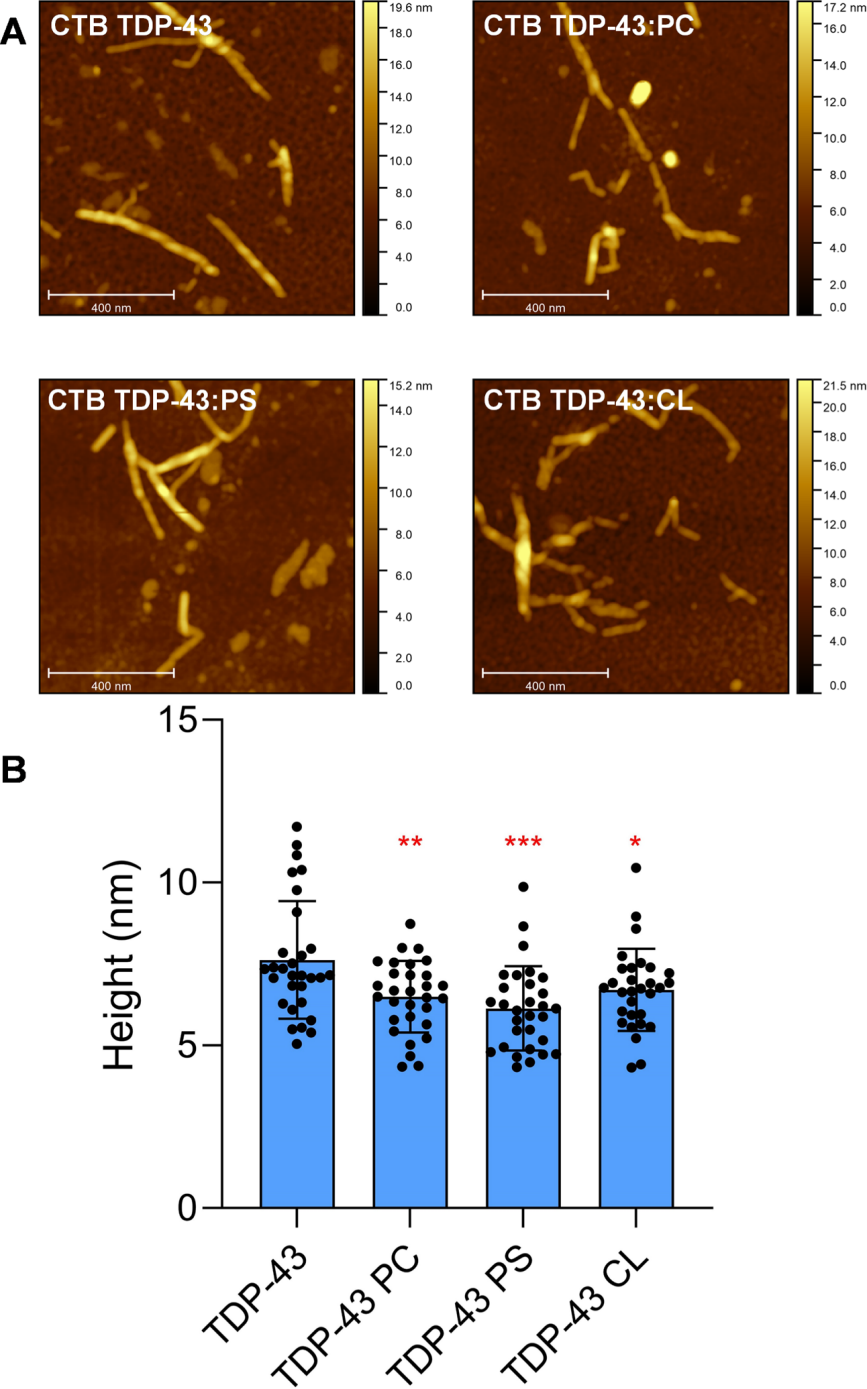
Morphological analysis of CTD TDP-43 aggregates. AFM images (A) and a bar graph (B) of height of CTD TDP-43 aggregates formed in the absence of lipids (CTD TDP-43) and in the presence of equimolar concentrations of LUVs composed of PC, PS, and CL. According to one-way ANOVA, **P < 0.01; ***P < 0.001.

Next, we used nano-IR to examine the secondary structures of these fibrils. Unlike conventional IR spectroscopy that probes the bulk volume of the sample, nano-IR allows for the precise positioning of the metallized scanning probe at the object of interest. Next, a pulsed tunable IR laser is used to cause thermal expansions in the fibrils, which are transmitted to the scanning probe and converted to the IR spectra. The acquired spectra possessed amide I and II bands, which originated from the peptide bond vibrations, Figure 4. The amide I band was fitted to elucidate the contribution of parallel β-sheet (1632 cm^–1^), unordered protein (1660 cm^–1^), and antiparallel β-sheet (1695 cm^–1^) in the fibrils formed by CTD TDP-43. We found that all analyzed fibrils had highly similar secondary structures composed of ∼35% parallel β-sheet, ∼45% unordered protein, and ∼20% antiparallel β-sheet. Thus, we could conclude that lipids did not alter the secondary structure of the CTD TDP-43 fibrils. It should be noted that the nano-IR spectrum acquired from CTD TDP-43:PC fibrils exhibited a band at 1730 cm^–1^, which originated from the C=O vibration of PC that was not evident in other acquired spectra, Figure 4. These results suggested that CTD TDP-43:PC fibrils possessed PC in their structure. It should be noted that the presence of lipids in fibrils had been reported for other protein aggregates.^36–38,42,48–50^ However, it was not commonly observed that lipids did not directly alter the secondary structure of amyloid fibrils.^36–38,42,48–50^

**Figure 4:**
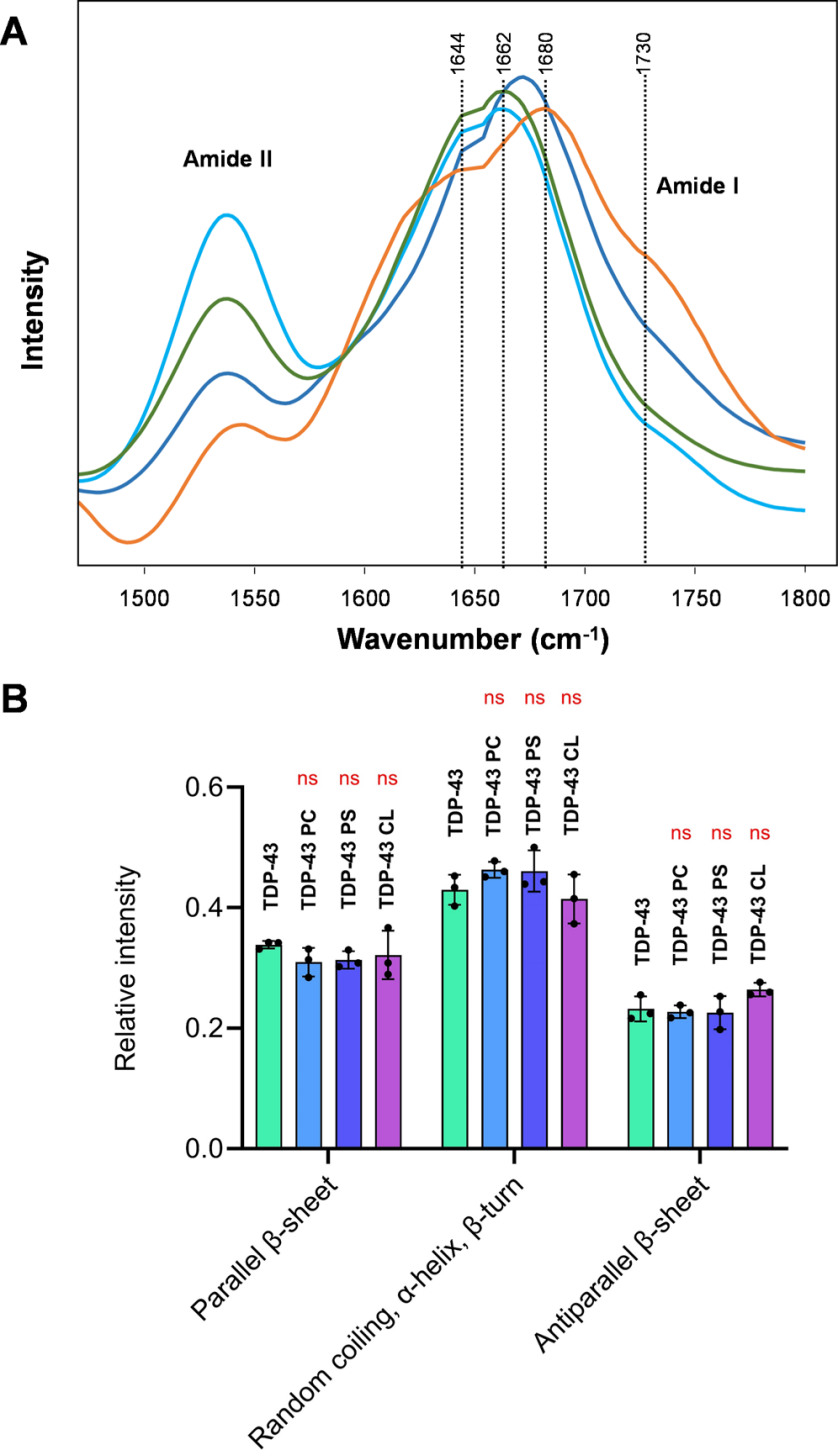
Structural analysis of CTD TDP-43 aggregates. Nano-IR spectra (A) and a bar graph (B) of amide I fitting of the spectra acquired from CTD TDP-43 aggregates formed in the absence of lipids (CTD TDP-43) and in the presence of equimolar concentrations of LUVs composed of PC, PS, and CL. Each curve shown in panels is the average of three replicates (*n* = 3). The graphical data are presented as the mean ± SEM. One-way ANOVA was used to examine statistical significance of the data; NS is nonsignificant differences.

We used rat dopaminergic cells to investigate the extent to which lipids alter the cytotoxicity of TDP-43 fibrils. Flow cytometry assays used in our study showed that all fibrillar samples exerted significant cytotoxicity to the neurons. This conclusion could be made from elevated levels of ROS observed in such cells after 48 h of exposition to the fibrils, Figure 5A. Our results also indicated that TDP-43 fibrils formed in the presence of PS were only slightly more toxic compared to fibrils formed in the absence of lipids. It should be noted that the cytotoxicity of TDP-43:PC and TDP-43:CL fibrils was the same as that of TDP-43 fibrils grown in the absence of lipids. Microscopic analysis of rat dopaminergic neurons exposed to TDP-43 fibrils grown in the presence and absence of lipids confirmed the flow cytometry findings. Specifically, we observed a high amount of ROS in such cells, which was not evident in the control and neuronal cells exposed to lipids themselves, Figure 5B.

**Figure 5:**
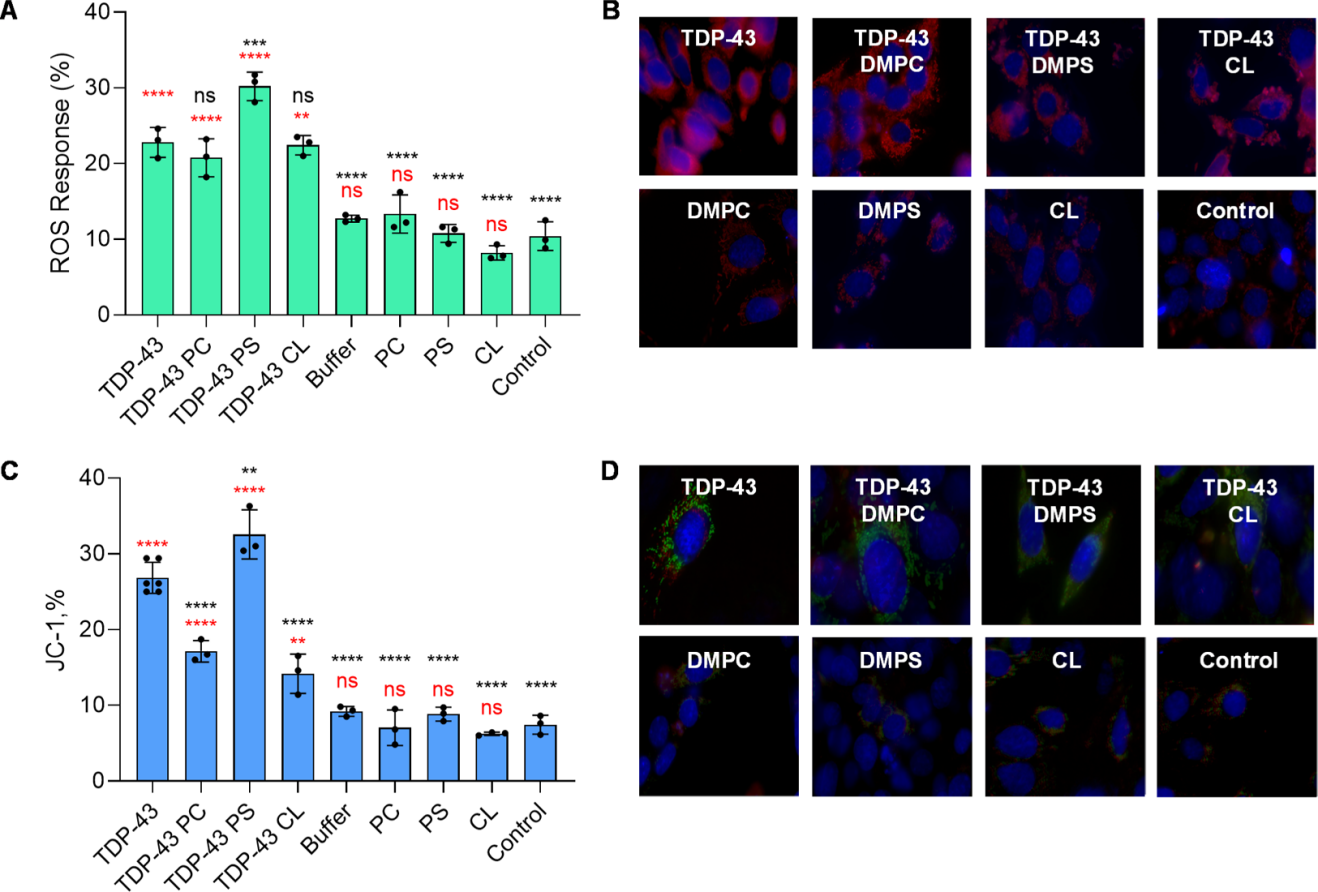
Lipids alter cytotoxicity of TDP-43 fibrils. Bar graphs of ROS (A) and JC-1 (C) toxicity assays of TDP-43 and TDP-43:PC. TDP-43:PS and TDP-43:CL buffers used to grow protein aggregates (Buff), as well as lipids themselves (PC, PS, and CL). For each of the results presented, three independent measurements were made. Red asterisks indicate statistical significance of all samples relative to the control; black asterisks indicate statistical significance of all samples relative to TDP-43 grown in the lipid-free environment. Each curve shown in panels is the average of three replicates (*n* = 3). The graphical data are presented as the mean ± SEM. According to one-way ANOVA*P < 0.05, **P < 0.01, ns—nonstatistical significance. Fluorescence microscopy images that show (B) ROS response (red) and (D) JC-1 monomer (green) and JC-1 polymer (red) fluorescence in N27 cells after 24 h exposition to protein aggregates and lipids. The blue fluorescence represents the nuclear fluorescence dye.

Utilizing the JC-1 assay, we determined the extent to which protein aggregates formed in the presence and absence of lipids damaged cell mitochondria. Our results showed that PC and CL decreased the toxicity of TDP-43 fibrils to the neuronal mitochondria, while this effect was not observed for PS, Figure 5C. We also found that TDP-43 fibrils formed in the presence of PS exerted only slightly stronger depolarization effects on the mitochondria compared to fibrils formed in the absence of lipids. These results indicated that lipids changed the cytotoxicity of TDP-43 fibrils to the neuronal mitochondria.

Microscopic analysis of the neurons exposed to TDP-43 fibrils reveals a substantial amount of depolarized mitochondria in the cells exposed to TDP-43 fibrils, formed in the lipid-free environment and in the presence of PC and PS, Figure 5D. Neurons exposed to TDP-43:CL fibrils, as well as lipids themselves, did not exhibit a significant amount of green fluorescence, which indicated depolarization of mitochondrial membranes. These results confirmed that lipids altered the cytotoxicity of TDP-43 fibrils to mitochondria present in dopaminergic neurons.

One could expect that TDP-43 aggregates may enter the cells via endocytosis, which results in the accumulation of protein aggregates in the cell endosomes. To test this hypothesis, we determined changes in the expression of two markers of endosomal damage: Chmp1 and Gal3. Chmp1 binds to membranes of damaged endosomes, activating the ESCRT-III complex involved in membrane repair.^51–53^ Fibril-induced endosomal damage also triggers leakage of luminal β-galactosides into the cytosol. Cytosolic Gal3 binds to exposed β-galactosides, initiating autophagy.^51,54^ Fluorescence imaging revealed significant changes in the expression of both Chmp1 and Gal3 in the cells exposed to TDP-43 aggregates grown in the presence and absence of lipids, Figure 6. These results indicated that TDP-43 aggregates damaged neuronal endosomes. We also found that lipids presented at the stage of protein aggregation did not substantially alter the degree to which TDP-43 aggregates damage endosomes in neurons. It should be noted that fibrils’ endocytosis and consequent endosomal damage exerted by TDP-43 aggregates may not be the only mechanism by which such fibrils enter the cell. One can expect that permeabilization of plasma membranes observed for other fibrils could be an alternative mechanism of TDP-43 aggregates’ cytotoxicity.^55^ Additional studies are required to fully understand the mechanisms of TDP-43 aggregates’ internalization by neurons, which are beyond the scope of the current work.

**Figure 6:**
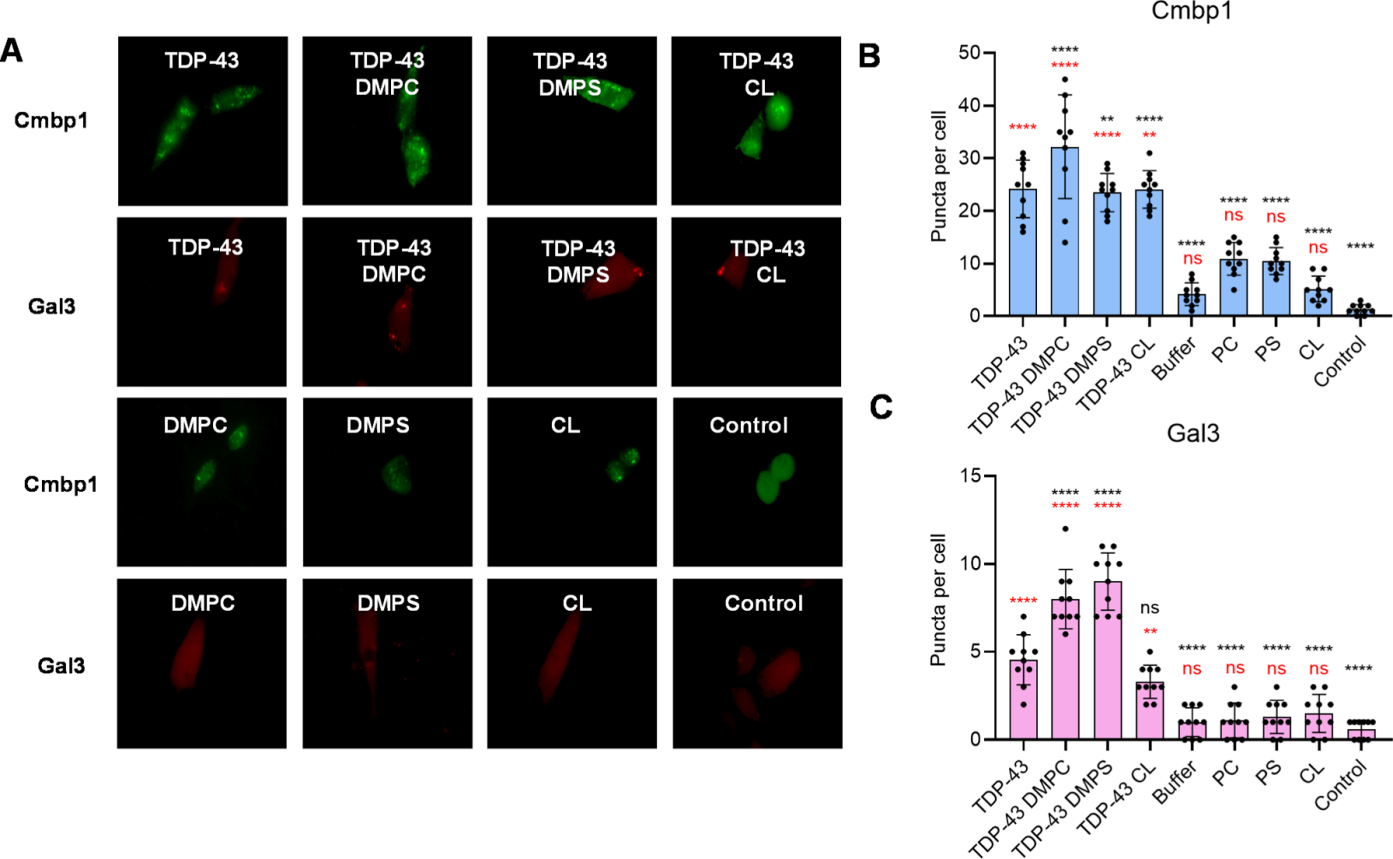
Fluorescent images (A) of N27 cells transfected with Chmp1 (green) and Gal3 (red) after the incubation with TDP-43 fibrils and lipids for 24 h. Bar graphs of fluorescent Chmp1 (B) and Gal 3 (C) puncta per cell observed in the cells. Red asterisks indicate statistical significance of all samples relative to the control; black asterisks indicate statistical significance of all samples relative to TDP-43 grown in the lipid-free environment. Each curve shown in panels is the average of three replicates (*n* = 3). The graphical data are presented as the mean ± SEM. According to one-way ANOVA, *P < 0.05, **P < 0.01, ***P < 0.001, ****P < 0.0001; ns—nonstatistical significance.

Next, we performed qPCR to reveal molecular mechanisms by which TDP-43 aggregates damaged neurons. Specifically, we investigated changes in the expression of PERK, ATF6, and XBP1, the key players in the unfolded protein response (UPR) of endoplasmic reticulum (ER), Figure 7.^56,57^ We found that TDP-43 fibrils grown in a lipid-free environment strongly upregulated the expression of XBP1, while TDP-43 fibrils formed in the presence of PC and PS inhibited the expression of ATF6 and XBP1. At the same time, TDP-43:PC and TDP-43:PS fibrils strongly upregulated the expression of the PERK activation pathway of UPR in the ER. We also found that TDP-43:CL fibrils upregulated the expression of all three main mechanisms of UPR in the ER. At the same time, lipids themselves did not cause a significant increase in the expression of PERK, ATF6, or XBP1. These results indicated that TDP-43 fibrils are associated with transcriptional changes consistent with UPR activation and ER stress. Our findings also showed that lipids uniquely altered the molecular mechanism by which TDP-43 fibrils induce cytotoxicity in neurons.

**Figure 7:**
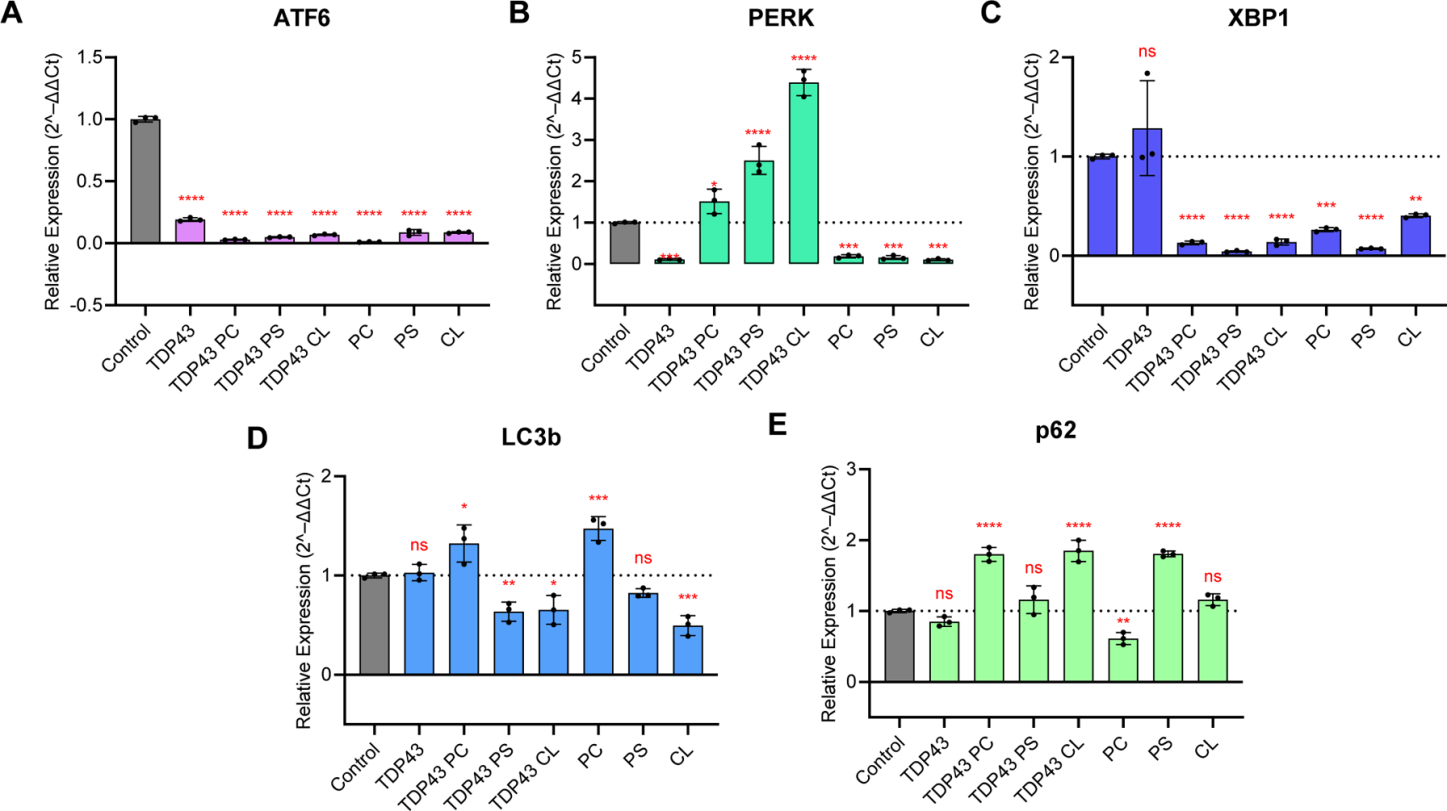
Bar graphs of ATF6 (A), PERK (B), XBP1 (C), LC3b (D), and p62 (E) expression in N27 neurons exposed to protein aggregates formed in the absence and presence of lipids, and cells exposed to lipids themselves. A control (buffer) was used as the calibrator to normalize the values to 1.0. Each number is the mean of three independent replicates (*n* = 3). The graphical data are presented as the mean ± SEM. According to one-way ANOVA, *P < 0.05, **P < 0.01, ***P < 0.001, ****P < 0.0001; ns—nonstatistical significance.

Our previous findings demonstrate that amyloid-induced toxicity is linked to dysregulation of cell autophagy.^58^ To analyze the extent to which the TDP-43 fibrils alter autophagy in neurons, changes in the expression of LC3b and p62 were quantified using qPCR, Figure 7. LC3b protein is required for autophagosome formation.^59–61^ LC3b also functions as an adaptor protein to recruit selective cargo to the autophagosome via interaction with cargo receptors.^62,63^ P62 is a ubiquitin-binding scaffold protein that colocalizes with ubiquitinated protein aggregates in many neurodegenerative diseases.^64,65^ We found that TDP-43 fibrils formed in the absence of lipids altered neither p62 nor LC3b. At the same time, TDP-43:PC fibrils strongly upregulated the expression of both of these proteins. TDP-43:CL only upregulated the expression of p62 while it inhibited the expression of LC3b. Finally, TDP-43:PS fibrils did not alter the expression of p62 but inhibited the expression of LC3b. These results indicated that TDP-43 fibrils formed in the presence and absence of lipids could alter cellular autophagy. Our findings also showed that lipids present at the stage of TDP-43 aggregation determine the extent to which TDP-43 fibrils damage cell autophagy. While our qPCR data indicate that TDP-43 fibrils formed in the presence of distinct phospholipids alter the transcript levels of UPR- and autophagy-related genes, we recognize that mRNA levels alone do not confirm protein expression or activation. Future studies incorporating Western blot or other protein-level validation will be essential to determine whether these transcriptional changes translate to functional pathway modulation.

## Conclusions

Our results show that lipids accelerate the aggregation of CTD TDP-43. Therefore, we can conclude that the CTD of TDP-43 is responsible for protein–lipid interactions. Furthermore, the acceleration effect is lipid specific. We found that anionic PS and CL exerted a much stronger acceleration effect on CTD TDP-43 compared to zwitterionic PC. These results indicate that an increase in the concentration of anionic lipids in lipid bilayers may trigger abrupt aggregation of CTD TDP-43. We also found that lipids change the morphology of CTD TDP-43 fibrils, although they do not alter the secondary structure of protein aggregates. Cell assays revealed that CTD TDP-43 fibrils formed in the presence of PC and CL were significantly less toxic to the cell mitochondria compared with CTD TDP-43 fibrils formed in the lipid-free environment. These results indicate that lipids present in the structure of CTD TDP-43 fibrils modulate the cytotoxicity of these protein aggregates. These results also suggest that PC and CL lipid vesicles might be used to lower the cytotoxicity of CTD TDP-43 to mitochondria and possibly decelerate the progression of ALS, FTD, AD, and LATE. It should be noted that the 279–360 CTD studied in the current work lacks RRMs and nuclear localization signal (NLS) that play an important role in protein–membrane interactions. Therefore, our results may not capture the more complex nature of TDP-43 interaction with lipids, as well as the effect of lipids on the protein aggregation kinetics and cellular trafficking. Finally, it is important to note that the cytotoxic effects of CTD TDP-43 aggregates were drawn from cell-based assays. Therefore, it is important to verify the observed results using living organisms.

## Supplementary Material



## Data Availability

The data that support the findings of this study are available from the corresponding author upon reasonable request.

## Notes

Scheme of experimental workflow and sequences of primers (PDF)
