# Supplementary material for: Elucidation of Molecular Mechanisms of Lipid-Altered Cytotoxicity of TDP-43 Fibrils
Source: ACS Chem Neurosci. 2026 Jan 29;17(4):823–32. doi: 10.1021/acschemneuro.5c00934 (PMC12921692; doi:10.1021/acschemneuro.5c00934)
Supplement: Supplementary file 1 [file cn5c00934_si_001.pdf]

Elucidation of Molecular Mechanisms of Lipid-Altered Cytotoxicity of TDP-43 Fibrils

Yana Purvinsh[1], Mikhail Matveyenka[1] and Dmitry Kurouski[*1]

1. Department of Biochemistry and Biophysics, Texas A&M University, College Station, Texas 77843, United States

Supporting Information

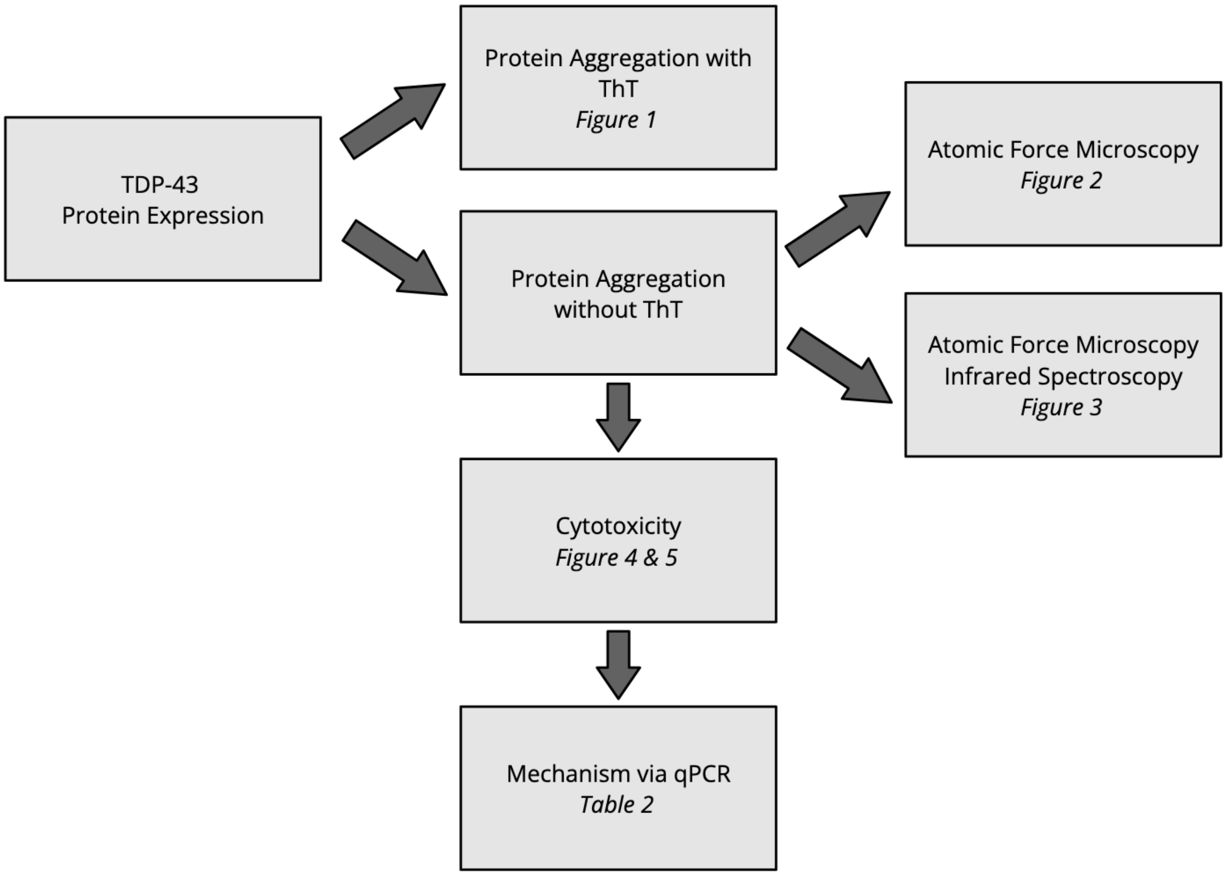

Figure S1. Experimental workflow of the performed studies.

Table S1. Sequence of primers used in the study.

Name of primers	Sequence
PERK Forward	5’- AGGATACGTGTCCCGATACC - 3’
PERK Reverse	5’- TCTCTGGTGGTGTTTCCAGC - 3’
ATF6 Forward	5’- CAAGACCGAAGATGTCCATTGTG - 3’
ATF6 Reverse	5’- ATCCTGGTGTCCATGACCTGA - 3’
XBP1 Forward	5’- CTGGTTGCTGAAGAGGAGG - 3’
XBP1 Reverse	5’- CATGGGGAGATGTTCTGGAG - 3’
P62 Forward	5’- AGAAGTGGACCCATCCACAG - 3'
P62 Reversed	5’- AGAAACCCATGGACAGCATC - 3'
LC3b Forward	5’- TTCTTCCTCCTGGTGAATGG - 3'
LC3b Reversed	5’- CTGGGAGGCATAGACCATGT - 3'
GAPDH Forward	5’ - GCACAGTCAAGGCTGAGAATG - 3’
GAPDH Reversed	5’ - TGGTGGTGAAGACGCCAGTA - ‘3’
